# 1850. Association between social vulnerability and *Streptococcus pneumoniae* antimicrobial resistance in US adults

**DOI:** 10.1093/ofid/ofad500.1678

**Published:** 2023-11-27

**Authors:** Salini Mohanty, Gang Ye, Charles Sheets, Nicole Cossrow, Kalvin Yu, Meghan White, Kenneth P Klinker, Vikas Gupta

**Affiliations:** Merck & Co., Inc, Rahway, New Jersey; Becton, Dickinson and Company (BD), Franklin Lakes, New Jersey; Becton, Dickinson & Company, Franklin Lakes, New Jersey; Merck & Co, Inc., Kenilworth, New Jersey; Becton, Dickinson and Company (BD), Franklin Lakes, New Jersey; Merck & Co., Inc., Rahway, New Jersey; Merck & Co., Inc., Rahway, New Jersey; Becton, Dickinson and Company (BD), Franklin Lakes, New Jersey

## Abstract

**Background:**

Antibiotic resistant infections affect more than 2.8 million people in the US each year and disproportionately impact those who are socially vulnerable. The objective of this study was to evaluate the association between *Streptococcus pneumoniae* (SP) antimicrobial resistance (AMR) and the CDC/ATSDR Social Vulnerability Index (SVI) in the US.

**Methods:**

Adult patients (≥ 18 years) with 30-day nonduplicate SP isolates from ambulatory/hospital settings from January 2011–December 2022 who had zip codes of residence reported were evaluated across 177 facilities in the BD Insights Research Database (Becton, Dickinson & Co., Franklin Lakes, NJ). Isolates were identified as SP AMR if they were intermediate or resistant to ≥ 1 macrolide, tetracycline, extended-spectrum cephalosporin, or penicillin. Isolates from adult patients with zip code of residence reported were matched with county-level CDC SVI data, and associations between SVI score (overall and by each theme) and SP AMR were evaluated using generalized estimating equations with repeated measurements within county to account for within-cluster correlations (Figure 1, Table 1).

**Figure:**
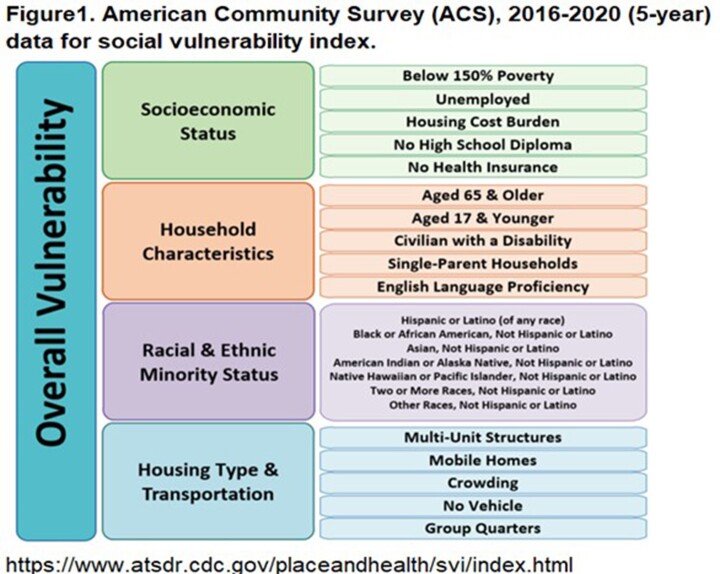

**Results:**

Of the 8,008 unique SP isolates linked to 574 US counties, the overall rate of SP AMR was 49.9%. A significant association between the socioeconomic status (SES) theme and SP AMR was detected with higher SVI scores (indicating greater social vulnerability) associated with greater risk of AMR. On average, a single decile increase in SES theme ranking score was observed to be associated with a 1.28% increased risk of AMR (95% confidence interval [CI], 0.61%, 1.95%; *P*=0.0002; Table 1). Similarly, a single decile increase in household characteristic theme ranking score was observed to be associated with a 0.81% increased risk in SP AMR (95% CI,0.13%, 1.49%; *P*=0.0197). However, no association between the racial and ethnic minority status theme, the housing type and transportation theme, and overall SVI score and SP AMR were observed.

**Figure:**
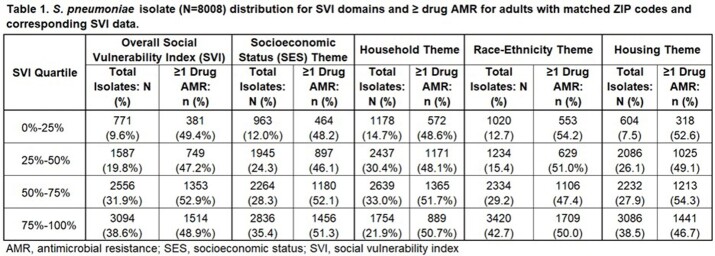

**Figure:**
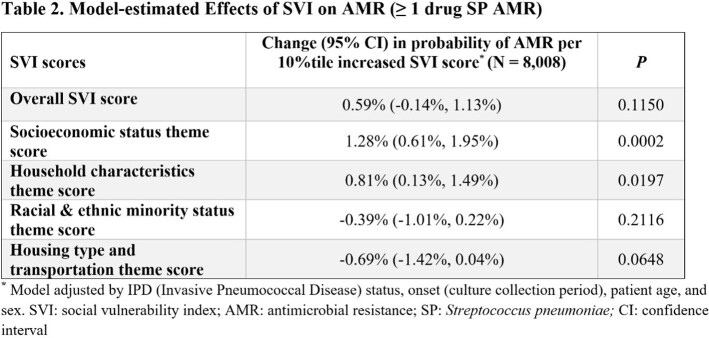

**Conclusion:**

Higher SES and household characteristic ranking scores (indicating greater social vulnerability) were observed to be associated with higher risk of SP AMR. These results contribute to evidence linking AMR to health disparities and inequities and suggest that AMR is impacted by social determinants of health.

**Disclosures:**

**Salini Mohanty, DrPH, MPH**, Employee of Merck & Co., Inc.: Stocks/Bonds **Gang Ye, PhD**, Becton Dickinson and Company: employee **Charles Sheets, MS**, Becton Dickinson: Data Analysis **Nicole Cossrow, PhD**, Merck: Stocks/Bonds **Kalvin Yu, MD, FIDSA**, BD: Stocks/Bonds **Meghan White, PharmD**, Merck: Employee|Merck: Stocks/Bonds **Kenneth P. Klinker, PharmD**, Merck & Co., Inc: Employee|Merck & Co., Inc: Stocks/Bonds **Vikas Gupta, PharmD**, Becton, Dickinson and Company (BD): Employee of BD|Becton, Dickinson and Company (BD): Stocks/Bonds

